# Exercise intervention during pregnancy can be used to manage weight gain and improve pregnancy outcomes in women with gestational diabetes mellitus

**DOI:** 10.1186/s12884-015-0682-1

**Published:** 2015-10-12

**Authors:** Chen Wang, Weiwei Zhu, Yumei Wei, Hui Feng, Rina Su, Huixia Yang

**Affiliations:** Department of Obstetrics and Gynecology of Peking University First Hospital, Xianmen Street No. 1, Xicheng District, Beijing, 100034 China; National Institute of Hospital Administration, Beijing, China

**Keywords:** Pregnancy, Gestational diabetes mellitus, Exercise intervention, Dietary intervention, Gestational weight gain, Preterm birth, Macrosomia, Low birth weight, Caesarean delivery

## Abstract

**Background:**

The study aimed to evaluate whether exercise intervention can be applied to pregnant women with gestational diabetes mellitus (GDM) for controlling gestational weight gain (GWG) and combating GDM-related outcomes.

**Methods:**

Retrospective six months analysis of 14,168 single pregnant women without diabetes from 15 hospitals in Beijing in 2013. Each participant’s demographic data, interventions condition and medical information were collected individually by questionnaires and relying on medical records. The level of statistical significance was set equal to 0.05.

**Results:**

2750 (19.4 %) pregnant women were diagnosed with GDM, 74.9 % of them received exercise intervention during pregnancy, and the starting time was 25.8 ± 3.7 gestational weeks. Women with GDM with exercise intervention (GDM-E) had the lowest BMI increase during late and mid-pregnancy than women with GDM without exercise intervention (GDM-nE) (2.05 ± 1.32 kg/m^2^ vs. 2.40 ± 1.30 kg/m^2^, *p* < 0.01) and non-GDM women (2.05 ± 1.32 kg/m^2^ vs. 2.77 ± 1.21 kg/m^2^, *p* < 0.01). Moreover, GDM-E group experienced a significantly lower risk of preterm birth (5.58 % vs. 7.98 %, *p* < 0.001), low birth weight (1.03 % vs. 2.06 %, *p* < 0.001) and macrosomia (9.51 % vs. 11.18 %, *p* > 0.05) than GDM-nE group. After including dietary factors in the analysis, women with GDM without either dietary or exercise intervention (GDM-nDnE) had the highest risk of preterm birth(OR = 1.64, 95 % CI, 1.14–2.36), while women with GDM with dietary intervention only (GDM-DnE) had the highest risk of low birth weight (OR = 3.10, 95 % CI, 1.23–7.81). However, women with GDM with both dietary and exercise intervention had the lowest rate of macrosomia.

**Conclusion:**

Exercise intervention is a suitable non-invasive therapeutic option that can be readily applied to manage weight gain and improve pregnancy outcomes in women with GDM.

## Background

Gestational diabetes mellitus (GDM) is a common complication of pregnancy. The prevalence of GDM has been reported to be as high as 16.1 %, and this rate is increasing worldwide [1].

A higher body mass index (BMI) before or in the first trimester of pregnancy and excessive gestational weight gain (GWG) during early and mid-pregnancy are both considered prominent early markers of GDM [2]. In women with both high pre-pregnancy BMI and excessive GWG, the risk of GDM increases by 2.2–5.9-fold [3]. Meanwhile, excessive GWG is one GDM-related complication [4]. Moreover, GDM and excessive GWG are strong predictors of mothers and offspring being overweight/obese a decade or more after the birth [5, 6]. Thus, there is a vicious cycle of excessive GWG, overweight/obesity and GDM. Certainly, pregnancy-related weight problems must be addressed and be paid adequate attention.

In addition, maternal overweight status and obesity [7], excessive GWG [8] and GDM [9] are all independently associated with an increased risk of adverse pregnancy outcomes, such as preterm birth, macrosomia and caesarean delivery, particularly when these three factors occur simultaneously [10]. American scholars determined that excessive GWG was the strongest contributor (33.3–37.7 %) to large-for-gestational-age neonates, more than GDM (2.0–8.0 %) or maternal pre-pregnancy overweight status and obesity (9.5–22.4 %) [11]. Similar results were published in two other studies [12, 13], which suggested that GWG outside the Institute of Medicine (IOM) recommendations after a diagnosis of GDM is associated with a 36–83 % increase in the risk of GDM-related perinatal adverse outcomes. Thus, we hypothesized that targeting gestational weight problems, particularly excessive GWG, which is a modifiable risk factor during pregnancy, would contribute to improving adverse GDM-related pregnancy outcomes.

Physical exercise is one of the best ways to control weight and stay healthy [14]. Currently, experts and obstetricians increasingly emphasize the role of exercise in preventing and managing GDM. A recent meta-analysis showed that high levels of activity before pregnancy (OR = 0.45, 95 % CI, 0.28–0.75) or in early pregnancy (OR = 0.76, 95 %, CI 0.70–0.83) were both significantly associated with a lower risk of GDM [15]. Previous observational studies also showed that women with GDM who undergo exercise intervention had superior blood glucose control and required a lower dose of insulin [16, 17].

However, studies that focus on pregnancy outcomes related to the use of exercise intervention are very limited. Therefore, to better understand the effects of exercise on GDM, the aim of this analysis was to examine whether exercise intervention is a suitable non-invasive therapeutic option that can be readily applied to pregnant women with GDM to control GWG and combat potential GDM-related adverse outcomes, including preterm birth, low birth weight, macrosomia and caesarean delivery, thus interrupting the GDM-centred vicious cycle.

## Methods

### Data source

This present analysis was part of a large retrospective study. In that study, based on the number of deliveries, 15 hospitals in Beijing were chosen as clusters by a systemic cluster sampling method; 15,194 pregnant women delivered from June 20th to November 30th, 2013, at these hospitals. The study was reviewed and approved by the Institutional Review Board of the First Hospital, Peking University (Reference number: 2013[578]). All participants provided written informed consent, and the ethics committee approved this consent procedure.

Women in our study were excluded for the following reasons: pre-existing diabetes, multiple births, and missing data on major items, such as 75 g oral glucose tolerance test (OGTT) results, birth weight, gestational age, delivery mode and whether women with GDM had exercise or dietary intervention during pregnancy.

### Data collection

A questionnaire was designed to obtain information by interviewing all the pregnant women and collecting medical records the day after they gave birth. The questionnaire had two primary parts as follows: a demographic information part that needed to be completed via a face-to-face interview in the patient’s room, and case data part that included information such as the woman’s weight at different gestational weeks, 75 g OGTT values, the neonatal’s birth weight, gestational age at delivery and similar parameters; the investigator was required to review and extract these data from each medical record. Certainly, information such as “whether you exercised or had dietary intervention during pregnancy” and “what time did you start to exercise or have dietary intervention” were self-reported and also need investigators to collect individually. Only women with GDM or diabetes mellitus (DM) were asked about exercise and dietary interventions, for these two items acted as two major methods in their treatment. Exercise intervention means sit less, take more steps, be more active, incorporate light and moderate PA as much as possible into their daily life et al., and diet intervention means reduce intake of sugar, eat more vegetables, reduce fat intake, and the total energy intake 1800 calories a day in all.

All the investigators in each hospital were trained before the survey. Each completed questionnaire was verified by an inspector. Data were coded and entered into a specially designed data software system that automatically checked for out-of-range values and logical mistakes. Moreover, all the questionnaires were entered independently by two persons and were verified by a third person.

### Definitions

1. GDM: The GDM diagnostic criteria followed the new criteria amended in August, 2014, in China, which recommend a diagnostic 75-g OGTT performed after the 24th week of gestation. GDM was diagnosed when any one value met or exceeded 5.1 mmol/L at 0 h, 10.0 mmol/L at 1 h, or 8.5 mmol/L at 2 h. Values of 7.0 mmol/L at 0 h or 11.1 mmol/L at 2 h should be always be diagnosed as DM [18].
2. GWG: Each participant’s GWG was represented by BMI increases, which were calculated using pre-pregnancy weight (within three months before pregnancy), mid-pregnancy weight (around the time of the 75-g OGTT), late-pregnancy weight (within the last week before giving birth) and height. Thus, we obtained the patients’ BMI increases between mid and pre-pregnancy, late and mid-pregnancy and late and pre-pregnancy. This necessitated that pre-pregnancy weight was self-reported, whereas mid and late-pregnancy weight were obtained from medical records.
3. BMI categories: The BMI categories were classified based on the following recommendation of the Group of China Obesity Task Force of the Chinese Ministry of Health: overweight, 24 ≤ BMI < 28 kg/m^2^; obese, BMI ≥ 28 kg/m^2^ [19].
4. Guidelines for GWG during pregnancy: underweight women should gain 12.5–18 kg; normal weight women, 11.5–16 kg; overweight women, 7–11.5 kg; and obese women, 5–9 kg [20].
5. Preterm birth: Gestational age at delivery less than 37 weeks.
6. Macrosomia: Foetal birth weight ≥ 4000 g, regardless of gestational age.
7. Low birth weight: Foetal birth weight < 2500 g with gestational age ≥ 37 weeks.
8. Physical activity during pregnancy: Light: No work or sitting while working, walking less than 60 minutes a day. Moderate: Activities that require moderate physical effort and make a pregnant woman breathe a little harder than normal (such as cooking, sweeping the floor, washing clothes, average daily commute longer than 60 minutes or walking more than 60 minutes a day, carrying light loads, or bicycling at a regular pace). High: Activities that require considerable physical effort and make a pregnant woman breathe much harder than normal (such as heavy lifting, aerobics, fast bicycling, dancing or swimming).
9. Statistical analysis.

We divided participants into the following three groups based on their diagnosis of GDM and whether they received exercise interventions during pregnancy: the non-GDM (normal) group, the GDM without exercise intervention group (GDM-nE) and the GDM with exercise intervention group (GDM-E). Because pregnant women with GDM should also receive dietary intervention during pregnancy, we further divided participants into subgroups to examine the role of exercise intervention separate from dietary intervention. The subgroups were the following: pregnant women with GDM without either dietary or exercise intervention (GDM-nDnE), pregnant women with GDM with dietary intervention only (GDM-DnE), pregnant women with GDM with exercise intervention only (GDM-EnD) and pregnant women with GDM with both dietary and exercise intervention (GDM-DE).

Data management was performed using the SPSS 13.0 statistical software package (Peking University Clinical Research Institute). Continuous variables are expressed as the mean ± standard deviation, and categorical variables are presented as numbers and percentages. Differences in the means between groups were evaluated using an independent samples T-test and analysis of variance (ANOVA). Pearson’s chi-square test was used for categorical variables. The level of statistical significance was set at 0.05. Odds ratios (ORs) were used as estimates of the effect of exercise intervention on improving pregnancy outcomes. Pregnant women in the Non-GDM group and GDM-DE group were used as the reference, and ORs with 95 % confidence intervals were calculated.

## Results

In total, 14168 pregnant women were recruited into this study. Among these women, 2750 (19.4 %) were diagnosed with GDM, of whom 2061 (74.9 %) stated that they had undergone exercise intervention during pregnancy. The mean time at which they began the exercise intervention was 25.8 ± 3.7 gestational weeks. The baseline characteristics of the study population are summarized in Table 1. Overall, the majority of the participants included in our study were between 22 and 35 years of age (89.8 %), and most had at least a high school education (81.7 %). Approximately 70.4 % of the participants were nulliparous, and 13.9 % reported a family history of diabetes. Based on their pre-pregnancy BMI and the 2009 IOM recommendations, 19.1 % were overweight or obese, and nearly half of the women (48.0 %) experienced excessive GWG. Age, pre-pregnancy BMI and a family history of diabetes in the current pregnancy were all higher among the pregnant women with GDM. The pregnant women’s physical activity during pregnancy is also shown in Table 1. Pregnant women with GDM with exercise intervention had the highest level of physical activity during pregnancy.

**Table 1:** Baseline characteristics of pregnant women enrolled in this study

	Total sample N = 14,168	Non-GDM N = 11,418	GDM-nE N = 689	GDM-E N = 2061
Age (years)*	28.12 ± 4.28	27.86 ± 4.21	28.70 ± 4.38	29.38 ± 4.40
Pre-pregnancy BMI* (kg/m^2^)	21.62 ± 3.29	21.36 ± 3.16	22.53 ± 3.69	22.71 ± 3.57
Exceeds recommended GWG	6303(48.0)	5092(48.3)	281(46.0)	930(47.3)
Family history of diabetes*	1969(13.9)	1392(12.2)	117(17.0)	460(22.3)
Physical labor during pregnancy*				
Light	10398(73.4)	8414(73.7)	530(76.9)	1454(70.5)
Moderate	3561(25.1)	2830(24.8)	154(22.4)	577(28.0)
High	75(1.2)	147(1.3)	4(0.6)	24(1.2)

Continuous variables were expressed as means ± SD and categorical variables were expressed as n (%)

*Abbreviations: BMI* body mass index (calculated as weight in kilograms divided by the square of height in meters), *GWG* gestational weight gain

*Indicates a significant difference among Non-GDM, GDM-nE and GDM-E group, *p* <0.001

Pre-pregnancy BMI and the BMI increase between mid and pre-pregnancy were both much higher in pregnant women with GDM compared with pregnant women without GDM (22.67 ± 3.60 kg/m^2^ vs. 21.36 ± 3.16 kg/m^2^ for pre-pregnancy BMI; 3.54 ± 2.02 kg/m^2^ vs. 3.31 ± 1.87 kg/m^2^ for the BMI increase between mid and pre-pregnancy, *p* < 0.001), especially in the GDM-E group (22.71 ± 3.57 kg/m^2^ for pre-pregnancy BMI; 3.59 ± 1.92 kg/m^2^ for the BMI increase between mid and pre-pregnancy). However, findings for the BMI increase between late and mid-pregnancy were quite the opposite. The BMI increases among the non-GDM, GDM-nE and GDM-E groups are shown in detail in Table 2. The BMI increases during late and mid-pregnancy were lower in the GDM-E group than in the GDM-nE group (2.05 ± 1.32 kg/m^2^ vs. 2.40 ± 1.30 kg/m^2^, *p* < 0.01) and the non-GDM group (2.05 ± 1.32 kg/m^2^ vs. 2.77 ± 1.21 kg/m^2^, *p* < 0.01); the total BMI increase, which was defined as the BMI increase from pre-pregnancy to late pregnancy, was lowest in the GDM-E group and highest in non-GDM group (GDM-E, 5.64 ± 2.22 kg/m^2^ vs. GDM-nE, 5.71 ± 2.55 kg/m^2^ vs. non-GDM, 6.05 ± 2.19 kg/m^2^).

**Table 2:** The BMI increases among the non-GDM, GDM-nE and GDM-E groups

	Total sample	Non-GDM	GDM-nE	GDM-E
Pre-pregnancy BMI (kg/m^2^)	21.62(3.29)	21.36(3.16)*^#^	22.53(3.69)*	22.71(3.57)^#^
Δ Mid-Pre BMI (kg/m^2^)	3.35(1.90)	3.30(1.87)*^#^	3.35(2.29)*^&^	3.59(1.92)^#&^
Δ Late-Mid BMI (kg/m^2^)	2.64(1.26)	2.77(1.21)*^#^	2.40(1.30)*^&^	2.05(1.32)^#&^
Δ Late-Pre BMI (kg/m^2^)	5.96(2.22)	6.04(2.19)*^*#^	5.71(2.55)*	5.64(2.22)^#^

Data expressed as mean (Standard Deviation)

*Abbreviations: BMI* body mass index, *Δ Mid-Pre BMI* BMI increase between mid and pre-pregnancy, *Δ Late-Mid BMI* BMI increase between late and med-pregnancy, *Δ Late-Pre BMI* BMI increase between late and pre-pregnancy

*Indicates a significant difference between Non-GDM group and GDM-nE group; ^#^Indicates a significant difference between Non-GDM group and GDM-E group; &Indicates a significant difference between GDM-nE group and GDM-E group. *^ #^ & *p* values were based on Independent Samples T test, *p* <0.05

Overall, 5.17 % of births occurred at a gestational age less than 37 weeks, and the mean gestational age was 39.0 ± 1.7 weeks. A total of 7.86 % of newborn infants were considered to have macrosomia, and 1.06 % of newborn infants had a low birth weight. Each outcome was worse among GDM pregnant women. However, Table 3 shows that compared with the GDM-nE group, women in the GDM-E group had an apparent lower risk of GDM-related adverse outcomes. Using the non-GDM group as a reference, women in the GDM-E group experienced a significantly lower risk of preterm birth than women in the GDM-nE group (5.58 %, OR = 1.14, 95 % CI, 0.93–1.40 vs. 7.98 %, OR = 1.68, 95 % CI, 1.26–2.24; *p* < 0.001) and of low birth weight (1.03 %, OR = 1.02, 95 % CI, 0.63–1.65 vs. 2.06 %, OR = 2.21, 95 % CI, 1.16–3.69; *p* < 0.001). The same trend was also observed in the risk of macrosomia, though the difference was not statistically significant (9.51 %, OR = 1.32, 95 %, 1.12–1.56 vs. 11.18 %, OR = 1.58, 95 % CI, 1.2–2.03; *p* > 0.05). There was no significant difference in the risk of caesarean delivery between the GDM-E group and the GDM-nE group (49.4 %, OR = 1.43, 95 % CI, 1.30–1.57 vs. 49.8 %, OR = 1.44, 95 % CI, 1.24–1.67; *p* > 0.05).

**Table 3:** Birth outcomes among the non-GDM, GDM-nE and GDM-E groups

	Preterm birth			Macrosomia (birth weight ≥ 4000 g)			Low birth weight (birth weight < 2500 g)			Caesarean delivery		
	n (%)	OR	95 % CI	n (%)	OR	95 % CI	n (%)	OR	95 % CI	n (%)	OR	95 % CI
Total Sample	732(5.17)			1113(7.86)			142(1.06)			6009(42.4)		
Non-GDM	562(4.92)	1.00	Reference	840 (7.36)	1.00	Reference	109(1.00)	1.00	Reference	4647(40.7)	1.00	Reference
GDM-nE	55 (7.98)	1.68	(1.26–2.24)	77(11.18)	1.58	(1.2–2.03)	13(2.05)	2.06	(1.16–3.69)	343(49.8)	1.44	(1.24–1.67)
GDM-E	115(5.58)	1.14	(0.93–1.40)	196(9.51)	1.32	(1.12–1.56)	20(1.03)	1.02	(0.63–1.65)	1019(49.4)	1.43	(1.30–1.57)

Pregnant women in the normal group were used as the reference

*Abbreviations: GDM* gestational diabetes mellitus, *OR* odds ratios, *CI* confidence interval

After including dietary factors in the analysis, the results remained the same, and they are shown in Tables 4 and 5. Because only 5 pregnant women stated that they had exercise intervention without dietary intervention, we excluded them. Women in the GDM-DE group had a higher pre-pregnancy BMI and a greater BMI increase between mid and pre-pregnancy but a smaller BMI increase between late and mid-pregnancy compared with the GDM-nDnE and GDM-DnE groups. Moreover, compared with the GDM-DE group, women in the GDM-nDnE group had a significantly increased risk of preterm birth (OR = 1.64, 95 % CI, 1.14–2.36), and women in the GDM-DnE group had a considerably increased risk of low birth weight (OR = 3.10, 95 % CI, 1.23–7.81). Furthermore, the rate of macrosomia was lower in the GDM-DE group compared to the GDM-DnE and GMD-nDnE groups (9.53 % vs. 10.34 % vs. 11.52 %, respectively).

**Table 4:** The BMI increases among the GDM-nDnE, GDM-DnE and GDM-DE groups

	GDM-nDnE N = 486	GDM-DnE N = 203	GDM-DE N = 2056
Pre-pregnancy BMI (kg/m^2^)	22.56(3.84)	22.45(3.31)	22.71(3.57)
Δ Mid-Pre BMI (kg/m^2^)	3.48(2.28)^#^	3.05(2.30)^#&^	3.59(1.92)^&^
Δ Late-Mid BMI (kg/m^2^)	2.50(1.31)^#*^	2.16(1.25)^#^	2.05(1.32)^*^
Δ Late-Pre BMI (kg/m^2^)	5.93(2.53)^#*^	5.18(2.55)^#&^	5.64(2.22)^*&^

Data expressed as mean (Standard Deviation)

*Abbreviations: BMI* body mass index, *Δ Mid-Pre BMI* BMI increase between mid and pre-pregnancy, *Δ Late-Mid BMI* BMI increase between late and med-pregnancy, *Δ Late-Pre BMI* BMI increase between late and pre-pregnancy

^#^Indicates a significant difference between GDM-nDnE and GDM-DnE group; *Indicates a significant difference between GDM-nDnE and GDM-DE group; &Indicates a significant difference between GDM-DnE and GDM-DE group. *^ #^ &p values were based on Independent Samples T test, *p* <0.05

**Table 5:** Birth outcomes among GDM-nDnE, GDM-DnE and GDM-DE groups

	Preterm birth			Macrosomia (birth weight ≥ 4000 g)			Low birth weight (birth weight < 2500 g)			Caesarean delivery		
	n (%)	OR	95 % CI	n (%)	OR	95 % CI	n (%)	OR	95 % CI	n (%)	OR	95 % CI
GDM-DE	115(5.59)	1.00	Reference	196(9.53)	1.00	Reference	20(1.03)	1.00	Reference	1017(49.5)	1.00	Reference
GDM-DnE	12(5.91)	1.06	(0.58–1.96)	21(10.34)	1.10	(0.68–1.76)	6(3.14)	3.10	(1.23–7.81)	108(53.2)	1.16	(0.87–1.55)
GDM-nDnE	43(8.85)	1.64	(1.14–2.36)	56(11.52)	1.24	(0.90–1.69)	7(1.58)	1.49	(0.63–3.54)	235(48.4)	0.96	(0.79–1.17)

Pregnant women in the GDM-DE group were used as the reference

*Abbreviations: OR* odds ratios, *CI* confidence interval

## Discussion

One important novel finding of our study was that exercise intervention during pregnancy significantly reduced BMI increases in women with GDM. The results stood after we combined the analysis of dietary intervention and exercise intervention.

The association between exercise intervention and GWG identified in our study was consistent with published literature. Shericka T. Harris et al. found that women who reported exercising ≥ 3 times a week were more likely to meet GWG recommendations (32.7 % vs. 18.7 %), and the OR of excessive GWG was lower (aOR = 0.43, 95 % CI, 0.24–0.78) than that for women who did not have the same intensity of exercise [21]. Likewise, Ronnberg A and colleagues found that total GWG was lower in the moderate-intensity (14.9 ± 3.8 kg) group compared with the low-intensity (15.3 ± 2.9 kg) and control groups (18.3 ± 5.3 kg). In addition, excessive GWG was prevented in 70 % of the women in the low-intensity group and 77 % of those in the moderate-intensity group [22]. A Chinese cohort study with 862 participants suggested that physically active pregnant women experience less weight gain during pregnancy, and the OR of excessive GWG decreased with an increased level of physical activity (*p* < 0.05) [23]. However, it should be noted that all of these studies were focused on healthy pregnant women. To the best of our knowledge, the participants in most existing studies of GWG and related issues were healthy overweight or obese pregnant women; therefore, our study is unique in its focus. Furthermore, we assessed the effect of exercise separately from that of dietary intervention.

Another main finding of our study was that exercise intervention during pregnancy could substantially combat GDM-related adverse outcomes, notably, preterm birth, low birth weight and macrosomia.

In our study, women with GDM had a higher risk of preterm birth, macrosomia and caesarean delivery compared to non-GDM pregnant women. However, we were delighted that exercise intervention could significantly reduce the risks of these adverse outcomes. Recently, Spanish scholars indicated that moderate exercise intervention performed over the second and third trimesters of pregnancy reduced the GDM-related risk of having a newborn with macrosomia and a caesarean delivery by 58 % and 34 %, respectively [24]. An earlier intervention study also showed that women who performed moderate aerobic activities three times per week during the entire pregnancy period had a lower frequency of macrosomia in newborn infants (6.0 % vs 12.5 %, *P* = 0.048) than those who performed little exercise [25].

Notably, in our study, women with GDM with only dietary intervention had a considerably increased risk of neonatal low birth weight; however, when the treatment was combined with exercise intervention as an adjunct therapy, other pregnancy outcomes improved, and the rate of low birth weight neonates significantly decreased.

Therefore, exercise must have had an extraordinary or independent effect on controlling the GWG of women with GDM and combating GDM-related adverse outcomes. As we mentioned above, scholars have already noted that excessive GWG after a diagnosis of GDM worsens GDM-related adverse pregnancy outcomes [13]. perhaps because GDM pregnant women who exercise have the best GWG control. Indeed, exercise might have some general basic mechanism of action [26].

In this study, we also found that the overall percentage of overweight or obese pregnant women in Beijing was 19.1 %, far higher than the percentage in 2009 [27]. This might be because China is now experiencing rapid economic, social and cultural changes. Therefore, an increasing number of women of reproductive age enter pregnancy overweight or obese, and this is associated with a heightened risk of GDM [28].

Accordingly, the rate of overweight/obese women in our study was as high as 30.7 % among pregnant women who developed GDM later, which is far higher than the overweight/obese rate among normal pregnant women (16.5 %). Similarly, the data from our study showed that pregnant women with GDM had much greater BMI increases between mid and pre-pregnancy, especially those who required exercise interventions later in pregnancy.

IOM guidelines provide specific pregnancy weight recommendations according to a woman’s pre-pregnancy BMI [20]. Though some published studies have been critical of the IOM recommendations [29] and have, in particular, questioned the guideline for GWG for obese pregnant women [30, 31], many studies have indicated that exceeding the IOM recommendations for GWG is associated with an increased risk of developing GDM [32]. Hedderson et al., noted that a woman’s risk of GDM increased with the rate of GWG, especially the rate of GWG in early pregnancy. Compared with GWG of less than 0.27 kg/week, GWG of 0.27–0.40 kg/week and more than 0.41 kg/week were associated with an increased risk of GDM (OR = 1.43, 95 % CI 0.96–2.14; OR = 1.74, 95 % CI 1.16–2.60, respectively) [32]. A more recent matched case–control study found that women with GDM gained significantly more weight by 24 weeks than those with normal glucose tolerance [33]. Similarly, a Chinese study with 90 GDM cases and 165 normal cases showed that compared with GWG of less than 0.28 kg/week, GWG of 0.28 kg/week or more was associated with an increased risk of GDM (OR = 2.03; 95 % CI 1.15–3.59) [34]. Additionally, all these studies emphasized that excessive GWG in the first trimester is the chief contributor to increasing a woman’s risk of GDM. Thus, this could be one reason to explain the result of our study that pregnant women with GDM with exercise intervention during pregnancy had the highest GWG between mid and pre-pregnancy. Perhaps these women have more severe and resistant blood glucose conditions.

Therefore, we are inclined to believe that exercise intervention initiated at the very beginning of pregnancy could decrease the risk of excessive GWG in the first and early second trimester and then possibly reduce the incidence of GDM among the entire population.

The American College of Obstetricians and Gynecologists (ACOG) recommends that pregnant women should exercise moderately for 30 minutes on most days of the week. However, very few women meet the minimum recommendations [35]. The data in our study also showed that only a small proportion of pregnant women performed moderate (25.1 %) to high (1.2 %) physical activity during pregnancy. The reasons for this might be that pregnancy is a very special period in a woman’s life; pregnant women are very careful about their actions during pregnancy [36], and they might not receive adequate advice and information concerning exercise from professionals [37]. Considering the prominent role of exercise during pregnancy in previous research and in our study, strengthening the research on pregnancy exercise-related issues, such as the optimal forms of exercise and intensity, will be of great value in improving pregnant women’s physical exercise level.

This study was conducted by trained staff who performed face-to-face interviews with pregnant women the day after they gave birth, and most of the items in the questionnaire were based on medical records. This method ensured the standardization of data collection. However, several limitations of this study should be noted. First, our study is a retrospective study, and items such as pre-pregnancy weight, physical activity during pregnancy and whether each woman had dietary or exercise intervention were self-reported; thus, it may contain recall bias. Second, information concerning the types of exercise intervention was not available in this study, so we could not examine the level and the duration of exercise intervention during pregnancy, nor could we specifically evaluate whether each woman really received an exercise intervention. However, to some extent, if pregnant women stated that they had exercise intervention during pregnancy, they should have at least been more active than not.

## Conclusion

Our findings provide some insights into the association of exercise intervention with GDM and GDM-related adverse outcomes. Pre-pregnant overweight/obese status and excessive GWG during pregnancy are both high-risk factors for GDM. Poor GWG control can aggravate GDM-related pregnancy outcomes. Exercise intervention is a good way to increase pregnant women’s physical activity during pregnancy, and it can also play an effective and particular role in managing the GWG of pregnant women with GDM; moreover, exercise might combat GDM-related adverse pregnancy outcomes. However, the overall level of physical activity among pregnant women is low. Above all, it is vital to call for action to improve pregnant women’s physical activity or increase their exercise levels during pregnancy.

## Abbreviations

GDM: Gestational diabetes mellitus
DM: Diabetes mellitus
GWG: Gestational weigh gain
BMI: Body mass index
OGTT: Oral glucose tolerance test
OR: Odds ratios
GDM-nE: GDM women without exercise intervention during pregnancy
GDM-E: GDM women with exercise intervention during pregnancy
GDM-nDnE: GDM women without either dietary or exercise intervention
GDM-DnE: GDM women with dietary intervention only
GDM-EnD: GDM women with execise intervention only
GDM-DE: GDM women with both dietary and exercise intervention
Δ Mid-Pre BMI: BMI increase between mid and pre-pregnancy
Δ Late-Mid BMI: BMI increase between late and med-pregnancy
Δ Late-Pre BMI: BMI increase between late and pre-pregnancy
